# Frontal Brain Asymmetry and Willingness to Pay

**DOI:** 10.3389/fnins.2018.00138

**Published:** 2018-03-13

**Authors:** Thomas Z. Ramsøy, Martin Skov, Maiken K. Christensen, Carsten Stahlhut

**Affiliations:** ^1^Neurons Inc., Holbæk, Denmark; ^2^Singularity University, Sunnyvale, CA, United States; ^3^Center for Decision Neuroscience, Department of Marketing, Copenhagen Business School, Frederiksberg, Denmark; ^4^Section for Cognitive Systems, Department of Informatics and Mathematical Modelling, Technical University of Denmark, Kongens Lyngby, Denmark

**Keywords:** willingness to pay, electroencephalography, neuroimaging, consumer neuroscience, neuromarketing, neuroeconomics

## Abstract

Consumers frequently make decisions about how much they are willing to pay (WTP) for specific products and services, but little is known about the neural mechanisms underlying such calculations. In this study, we were interested in testing whether specific brain activation—the asymmetry in engagement of the prefrontal cortex—would be related to consumer choice. Subjects saw products and subsequently decided how much they were willing to pay for each product, while undergoing neuroimaging using electroencephalography. Our results demonstrate that prefrontal asymmetry in the gamma frequency band, and a trend in the beta frequency band that was recorded during product viewing was significantly related to subsequent WTP responses. Frontal asymmetry in the alpha band was not related to WTP decisions. Besides suggesting separate neuropsychological mechanisms of consumer choice, we find that one specific measure—the prefrontal gamma asymmetry—was most strongly related to WTP responses, and was most coupled to the actual decision phase. These findings are discussed in light of the psychology of WTP calculations, and in relation to the recent emergence of consumer neuroscience and neuromarketing.

## Introduction

How do we decide how much we are willing to pay for a product? What are the basic mechanisms underlying such computations? In economics and the consumer sciences, one central concept in this regard is the Willingness To Pay (WTP), defined as the maximum amount of resources that a consumer is willing to give up in exchange for an object or service being sold (O'Brien and Viramontes, 1994; Homburg et al., 2005). Research has linked WTP to the evaluation to subsequent consumer motivation and real consumption choice in as diverse consumer choices as health services (Olsen and Smith, 2001), organic and healthy food products (Misra et al., 1991), country of origin effects (Loureiro and Umberger, 2003), and products that are either seen as environmentally friendly or otherwise ethically sound (Ozanne and Vlosky, 1997; Vlosky et al., 1999; De Pelsmacker et al., 2005).

Despite the study of WTP in such a variety of situations, little is known about the underlying neural or psychological processes of these calculations. Recent calls for an improved understanding of the basic mental mechanisms underlying WTP calculations have proposed to include neurobiological explorations of how the brain calculates values and instigates choice behavior. Still, in spite of the rapidly expanding research fields of neuroeconomics (Rolls, 2000; Camerer et al., 2005, 2016; Kenning and Plassmann, 2005; Rustichini, 2005; Kable and Glimcher, 2009; Wilhelms and Reyna, 2014; Levy and Glimcher, 2016), neuromarketing or consumer neuroscience (Ariely and Berns, 2010; Fisher et al., 2010; Plassmann et al., 2012, 2015; Smidts et al., 2014; Ramsøy, 2015; Hsu, 2017; Lee et al., 2017). In these multidisciplinary efforts, any exact understanding of the neural or psychological mechanisms underlying WTP calculations is still woefully lacking (Plassmann et al., 2012). In a study by Plassmann et al. (2007) it was found that activation in both the right ventromedial prefrontal cortex (vmPFC) and the right dorsolateral PFC (dlPFC) demonstrated a significant relationship to subjects' WTP. However, as the researchers noted, the exact role of the OFC and dlPFC in the calculation of WTP could not be determined. Notably, the authors speculated that based on the different connectivity that the two brain regions have, the multisensory nature of OFC could point to a role in the immediate valuation of items (Rolls, 2000, 2004), while the dlPFC could be more involved in the execution of choice behavior (Petrides and Pandya, 1999). More recent accounts also support a dissociation between value calculation and choice execution (and choice conflict) between the OFC and regions such as the ACC and dlPFC (Plassmann et al., 2010; Rushworth et al., 2012).

The study reported here was set up to explore the possible role of prefrontal hemispheric differences in computing WTP. The notion that the computation of WTP may rely on a diverse contribution from the two hemispheres is rooted in a growing body of work demonstrating a prefrontal asymmetry in relationship to approach and avoidance behaviors. The main findings emerging from this research are that approach behaviors are related to a relative stronger engagement of the left PFC compared to the right PFC (Pizzagalli et al., 2005), and that such effects are mainly due to motivation and not valence (Harmon-Jones and Allen, 1998). The left-hemispheric dominance for approach behaviors has also been suggested in studies of consumer choice (Ravaja et al., 2012), as well as advertising (Ohme et al., 2009, 2010). However, there is still conflicting evidence with regard to the hemispheric asymmetry model (Spielberg et al., 2008), and studies on the inverse effect of stronger relative engagement of the right compared to left PFC in avoidance behavior has been less consistent (Harmon-Jones and Allen, 1998). Interestingly, in the Plassmann et al. study we cited above (Plassmann et al., 2007), although the subjects were not explicitly tested for prefrontal asymmetry, the increased activations in the vmPFC and dlPFC were exclusively located in the right hemisphere.

In the present study, by asking subjects to watch images of different products while prefrontal asymmetry was assessed using electroencephalography (EEG), we find that a prefrontal laterality index obtained during passive product viewing is highly related to subsequent WTP reports. Notably, while prior EEG studies have focused on prefrontal asymmetry effects using alpha frequency, our results show that gamma frequency and beta frequency can have equal or even stronger relationship to choice behavior.

## Materials and methods

Sixteen women (age range 19–51, mean/std = 27.1/8.2, all right handed) were recruited using both online (www.forsoegsperson.dk and www.videnskab.dk) and direct recruitment procedures. The study reported here was part of a larger cohort study on compulsive consumption (*n* = 63), but for the present study we only included subjects who did not meet diagnostic criteria for compulsive consumption, or the preclinical compensatory consumption stage, as assessed by the Compulsive Buying Scale (Faber and O'Guinn, 1992). All subjects read and signed an informed consent, and were initially informed and trained with the experimental procedure. The study was approved by the local ethics committee (at Copenhagen Business School) and abided to the regulations of the Declaration of Helsinki.

### Experimental design

**Figure 1:**
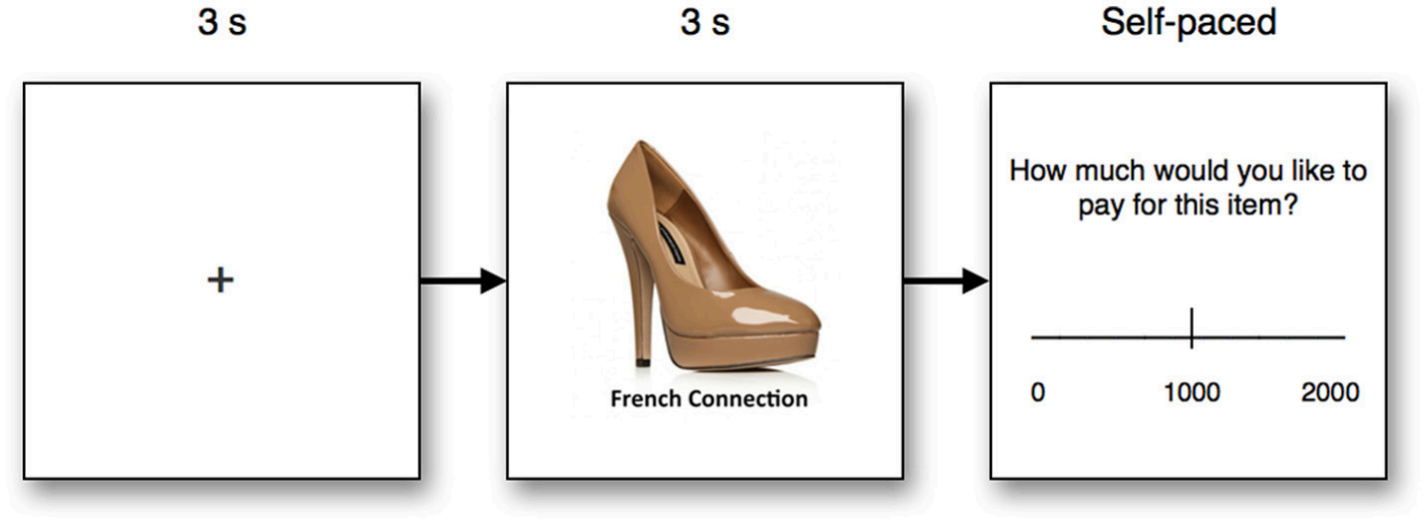
Experimental design. Subjects first saw a fixation cross for 3 s, followed by a product with accompanying brand information for 3 s. The product image was accompanied by the brand name of the product. Finally, subjects chose the amount of money (Danish kroner) they were willing to pay for the product using a visual analog scale, and in a self-paced manner.

Subjects were placed in front of a screen running with a 1,920 × 1,200 pixel screen resolution, and were placed at an approximate distance of 60 cm from the screen. During the test, subjects first saw a fixation cross for 3 s, followed by an image of a product from one of four categories; bags, clothes, women's shoes, and fast-moving consumer goods (FMCG). The individual product was shown for 3 s, after which asked to report how much they would like to pay for the product, using an on-screen visual analog scale ranging from zero to 2,000 Danish Kroner (≈$330). The experimental design is illustrated in Figure 1. In total, each participant was exposed to 40 trials, 10 trials in each product category (bags, clothes, FMCG, shoes). To increase the external validity of the test, subjects were instructed that the choices from two of the subjects from the cohort would be randomly selected and given 1,500 DKK each, and that five of each subject's choices would be randomly selected, and the product receiving the highest bid of those five would be realized. Should the highest bid not amount to 1,500 DKK, they would be paid the remaining amount in cash. This meant that subjects were motivated to optimize their product choices, which allowed us to better estimate the actual WTP, instead of subjective estimates of WTP. While this approach allowed the opportunity for participants to employ certain decision strategies such as selecting the minimally possible difference in price to signify preference (e.g., 2 DKK for the preferred item, relative to 1 DKK for non-preferred items) and retain the remaining amount in cash, no such strategy was found in the WTP choices made. In total, 640 observations were made (16 participants, 40 WTP decisions each).

Since the WTP scores were not normally distributed (Kolmogorov-Smirnov-Lilliefors test *D* = 0.276, *p* < 0.01), we chose to log transform the WTO score to achieve normal distribution of the WTP data, thus providing a logWTP score (producing a normal distribution of KSL test *D* = 0.071, *p* = 0.201), which we used in all analyses.

### Neuroimaging

**Figure 2:**
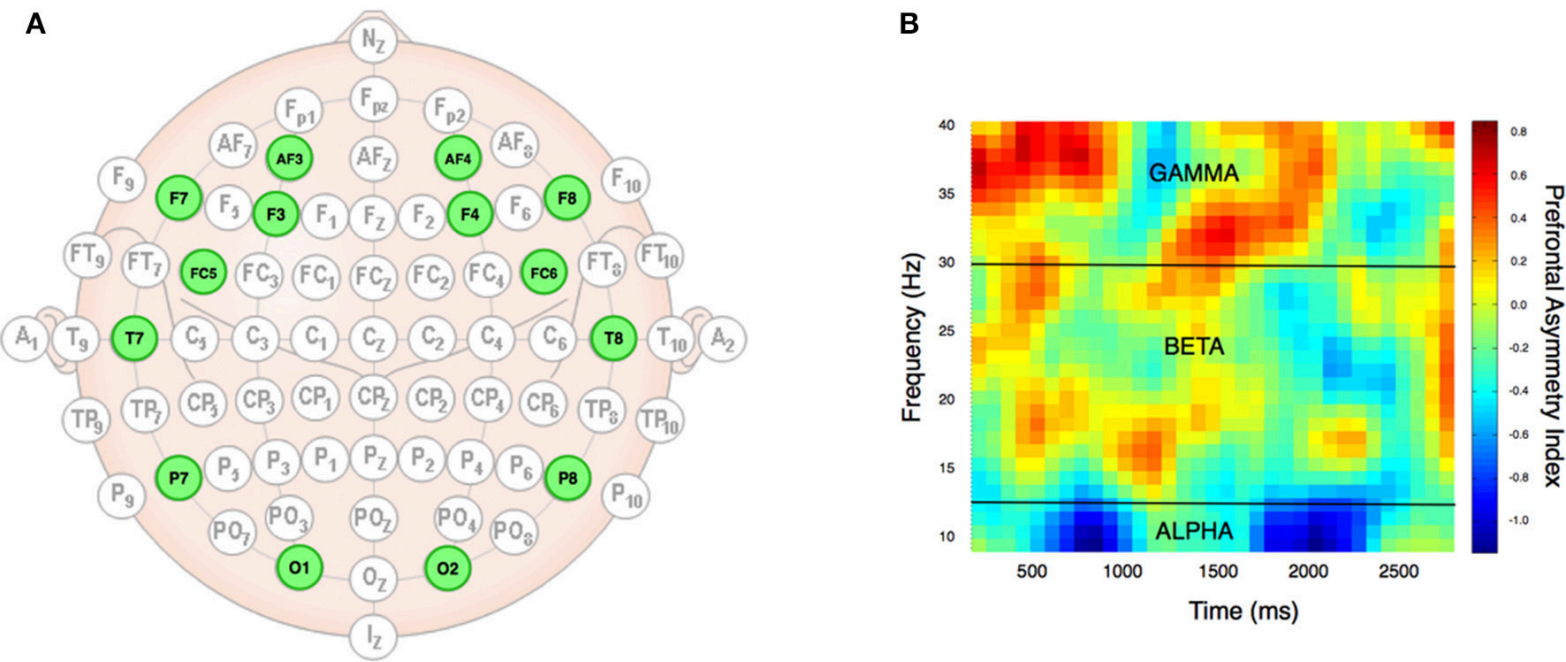
EEG setup and data types. The electrodes were positioned bilaterally according to the 10/20 system **(A)** at 14 specific locations (highlighted in green). From the raw EEG data, Fourier transformation provided alpha, beta, and gamma frequency bands, which allowed analysis of prefrontal asymmetry over time in these three different frequency domains, as exemplified by data from a single subject **(B)**. The **(B)** plot was achieved by running the PAI analysis on the frequency bands from 10 to 40 with an increment of 1 Hz (y-axis), and over time with a range from 100 to 3,000 ms with increments of 100 ms (x-axis). The heat map indexes the prefrontal asymmetry normalized as z-scores. Yellow to red colors denote positive PAI score, while cyan to blue colors denote low PAI scores. Black lines indicate frequency band borders of alpha, beta, and gamma.

Neural responses were recorded using a wireless 14 channel headset (Emotiv EPOC Inc.) with a sampling rate of 128 Hz (bandwidth = 0.2–43 Hz, digital notch filters at 50 and 60 Hz, and with built-in digital 5th order Sinc filter) and electrodes positioned at AF3, F7, F3, FC5, T7, P7, O1, O2, P8, T8, FC6, F4, F8, AF4 (the 10–20 system, see Figure 2). The headset was connected to a PC running Windows 7 and transmitted data wirelessly to a USB receiver module. Stimulus presentation and data collection for behavioral responses and neuroimaging data was performed using Attention Tool 4.5 (iMotions, www.imotionsglobal.com).

Offline data processing was carried out using the EEGLAB toolbox in Matlab. Baseline correction was performed on the entire recording to minimize potential drifts of electrodes. To avoid influence of bad channels affecting the analysis, the quality of each electrode was evaluated during the whole recording. Channel quality was provided for each sample with the Attention Tool 4.5 software with a categorization between 1 and 4 corresponding to no connection (poor) to good contact, respectively. In the analysis we declared an electrode as bad if the channel quality did not fulfill a criterion of having minimum 95% good channel quality during the whole recording. Bad channels were rejected from further analysis. In this study, we observed an average signal quality of 84.42 ± 0.20 st.dev percent, with some channels providing 100% average signal quality (AF3, F7, T7, and F8) while other channels produced the lowest average signal quality, and with large variation across individuals (average ± st.dev: O1 = 46.92 ± 49.91%, O2 = 41.06 ± 49.19%). The channels of most interest for this study produced signal quality well above acceptable levels (F3 = 82.40 ± 38.08%, F4 = 94.13 ± 23.50%). Only participants with valid data for the F3 and F4 electrodes were used in the study.

Power spectra were calculated on each electrode using windows of 50 samples (i.e., 390 ms) with a 80% overlap and a frequency resolution of 1 Hz. The power spectra were reduced to frequency bands in accordance with the alpha, beta, and gamma frequency bands, defined as alpha [8–13 Hz], beta [13–25 Hz], and gamma [25–40 Hz]. Each band was calculated as a summation of the total power within the band.

For each electrode alpha, beta and gamma frequencies were included in the analysis. The prefrontal asymmetry index (PAI) was calculated by subtracting the values from the AF4 (right prefrontal) electrode from the AF3 (left prefrontal) electrode, and divided by the sum of the two electrodes. This is illustrated by the following formula:

$$\frac{\log\;\left(AF_{3}\right)-log\;\left(AF_{4}\right)}{\log\;\left(AF_{3}\right)+log\;\left(AF_{4}\right)}$$

This means that for gamma (PAI_γ_) and beta (PAI_β_) frequencies, more positive values would be indicative of stronger engagement of the left PFC, and that more negative values would be related to relative stronger engagement of the right PFC. The frontal asymmetry was corrected for overall brain engagement by dividing the F3/F4 ratio on the sum of both channels (F3+F4). The alpha frequency has been linked to inhibitory brain function and is thus assumed to be negatively related to neural activation levels (Başar et al., 2001; Palva and Palva, 2007). The alpha measure (PAI_α_) has an inverse sign to that of gamma and beta; thus, more negative values would be indicative of relative stronger engagement of the left PFC.

We first ran a mixed model with logWTP as the dependent variable, with the prefrontal asymmetry for each frequency (PAI_α_, PAI_β_, and PAI_γ_) for the aggregate response of the 3 s product viewing time as independent variables, and with subject as random factor. To increase the specificity of the prefrontal activation, all alpha, beta, and gamma electrode values (F7, F3, FC5, T7, P7, O1, O2, P8, T8, FC6, F4, and F8) were modeled as regressors of no interest. This was imposed to increase the specificity of the frontal asymmetry measure.

Following this, we tested whether the inclusion of product type would improve the overall explanatory value of the model. To this end, we ran a new mixed model analysis with logWTP as the dependent variable, and with product category (FMCG, clothing, shoes, bags), prefrontal asymmetry (PAI_α_, PAI_β_, and PAI_γ_) and the category^*^PAI interactions as independent variables, again with subject as random factor.

To record and correct for the degrees of freedom used in denominator of each test, we use the term “Degrees of Freedom for the Denominator” (DFDen). The DFDen is calculated using the Kenward–Roger first order approximation (Kenward and Roger, 1997), and shows the denominator degrees of freedom for the effect test (the degrees of freedom for error).

We were interested in testing whether the relationship between prefrontal asymmetry and WTP would be modulated by stimulus duration (and, consequently, the time to decision). We therefore ran a mixed model analysis with logWTP (hereafter WTP) as the dependent variable, and with time, prefrontal asymmetry (PAI_α_, PAI_β_, and PAI_γ_) and the time^*^PAI interactions as independent variables, and with subject as random factor.

Finally, to test for other types of EEG responses related to WTP, we tested the relationship between all electrodes in the alpha, beta and gamma frequency bands, and their relationship to WTP, using a mixed model where logWTP was the dependent variable, each electrode alpha, beta, and gamma values were used as independent variables, subject was used as a random factor, and product category as a regressor of no interest.

## Results

Products received an average WTP of 202.25 Danish Kroner (DKK), but also demonstrated a large variance (STD = 339.36; range = 0:1627.9 DKK). There was a significant difference between the four product categories in logWTP score (*R*^2^ = 0.452, *F* = 41.1, *p* < 0.0001) that was driven by a lower logWTP for FMCG (4.51 ± 0.01) than the other product categories (bags = 6.33 ± 0.01; clothing = 6.03 ± 0.01; shoes = 5.98 ± 0.01).

In our first mixed model analysis, we tested the effect of prefrontal asymmetry during product viewing on subsequent WTP. The overall model was significant (*R*^2^ = 0.267, RMSE = 0.999, *F* = 5.37, *p* = 0.0013). As Table 1 shows, only PAI_γ_ showed a statistically significant effect, while PAI_β_ was trend significant, and PAI_α_ did not produce a significant result.

**Table 1:** Main effects of laterality on WTP.

**Term**	**Estimate**	**Std Error**	**DFDen**	***T***	***p***
Intercept	6.052	0.166		36.55	<0.0001^*^
logALPHA	−0.108	0.083	172.9	−1.31	0.1914
logBETA	0.161	0.096568	281.7	1.67	0.0967
logGAMMA	0.165	0.05665	158.1	2.92	0.0038^*^

*Independent relationship between each PAI measure and subsequent WTP for products. Asterisk denotes significant effects at p < 0.01*.

**Figure 3:**
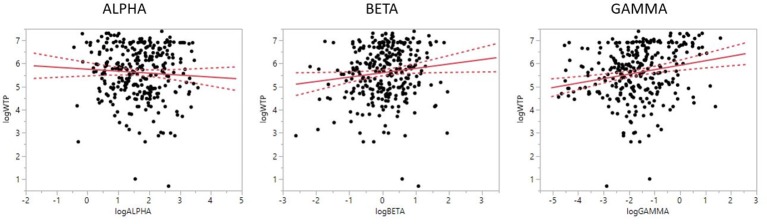
Prefrontal laterality for alpha, beta, and gamma frequencies and WTP. Relationship between prefrontal laterality and WTP. While the alpha laterality index showed no significant relationship to WTP, gamma (and a trend for beta) demonstrated a positive relationship. Negative numbers on the laterality index (x-axis) indicate stronger right than left engagement. Please note that the higher alpha frequency band activation is inversely related to the active engagement of a brain region. Solid line indicates mean value, dotted line indicates 95% CI.

Looking at the individual effects, and as shown in Figure 3, (PAI_γ_ *R*^2^ = 0.270, RMSE = 0.996, estimate = 0.161, *t* = 2.92, *p* = 0.0038) was positively related to WTP, i.e., more positive values were related to higher WTP. Similarly, PAI_β_, although not reaching statistical significance, showed a trend for a positive relationship to WTP (*R*^2^ = 0.273, RMSE = 0.993, estimate = 0.161, *t* = 1.67, *p* = 0.0967), which means that stronger engagement of the left PFC in beta frequency band was related to higher WTP. Finally, PAI value in the alpha range (*R*^2^ = 0.276, RMSE = 0.992, estimate = −0.085, *t* = −1.07, *p* = 0.2858) was negatively related to WTP, but did not produce a significant result. Although not significant, this result lends support to the frontal asymmetry and WTP, as more negative PAI_α_ values (i.e., stronger relative engagement of the left PFC, due to the inverse aspect of the alpha frequency relative to brain activity) were related to higher WTP scores.

When analyzing PAI_α_, PAI_β_, and PAI_γ_ independently, the relationship was significantly for PAI_β_ and PAI_γ_ only (alpha: *t* = −1.07, *p* = 0.286; beta: *t* = 2.11, *p* = 0.036; gamma: *t* = 3.55, *p* = 0.0004). Further analyses into the relationship between the three asymmetry scores demonstrated the following interrelationships: correlation between alpha and beta: *r* = 0.286, *p* < 0.0001; alpha and gamma: *r* = −0.132, *p* = 0.0009; beta and gamma: *r* = 0.202, *p* < 0.0001). In effect, this suggests that we find a positive relationship between PAI_α_ and PAI_β_, and between PAI_β_ and PAI_γ_, while we find a negative relationship between PAI_α_ and PAI_γ_.

In the second analysis, the main effect of product category and interactions with the PAI scores were included in the mixed model analysis. The overall model was highly significant (*R*^2^ = 0.641, *F* = 19.15, *p* < 0.0001). As shown in Table 2, only the category^*^PAI interactions were significantly related to subsequent WTP. Notably, when category was included in the analysis, PAI_β_ showed neither a significant main effect or interaction with category, while PAI_γ_ showed both a main effect and interaction effect with category. Looking further into this interaction effect, we find that PAI_γ_ is significantly positively related to logWTP for bags (*t* = 2.38, *p* = 0.018) and shoes (*t* = 1.66, *p* = 0.845), and that for FMCG there is a significant negative relationship (*t* = −2.80, *p* = 0.006), while clothing did not show a significant relationship (*t* = −0.19, *p* = 0.845).

**Table 2:** Effects of interaction between frequency band and product category.

**Source**	**DF**	**DFDen**	***F***	***p***
PAIα	1	176.2	0.8164	0.3670
PAIβ	1	267.4	0.7128	0.3993
PAIγ	1	172.1	12.2084	0.0006^*^
Category^*^ PAI_α_	3	262.4	0.5146	0.6726
Category^*^ PAI_β_	3	260.8	1.2099	0.3065
Category^*^ PAI_γ_	3	258.3	4.3411	0.0052^*^
Category	3	260.6	77.5436	<0.0001^*^

*Interaction effects (denoted by asterisk) between PAI measures and category, showing that prefrontal asymmetry in the all ranges interacts with product category*.

A control analysis was run to test the added value of neuroimaging data on product category in explaining WTP. We first ran a mixed model with product category alone, which yielded a significant model (*R*^2^ = 0.452, *F* = 79.04, *p* < 0.0001). We then included the full model with EEG scores but without interaction effects, and found that the overall explanatory value of the model increased (*R*^2^ = 0.641, *F* = 19.15, *p* < 0.0001), suggesting that the addition of EEG provided an increase in explanatory power of WTP. To test whether neuroscience data provided a significantly improved explanatory power, we ran a Chow *F*-test (Chow, 1960) on the pseudo-*R* values, which yielded a significant result (*F* = 1.73, *p* = 0.0464), suggesting that there was a significant additional explanatory value of adding the EEG data to the model.

**Figure 4:**
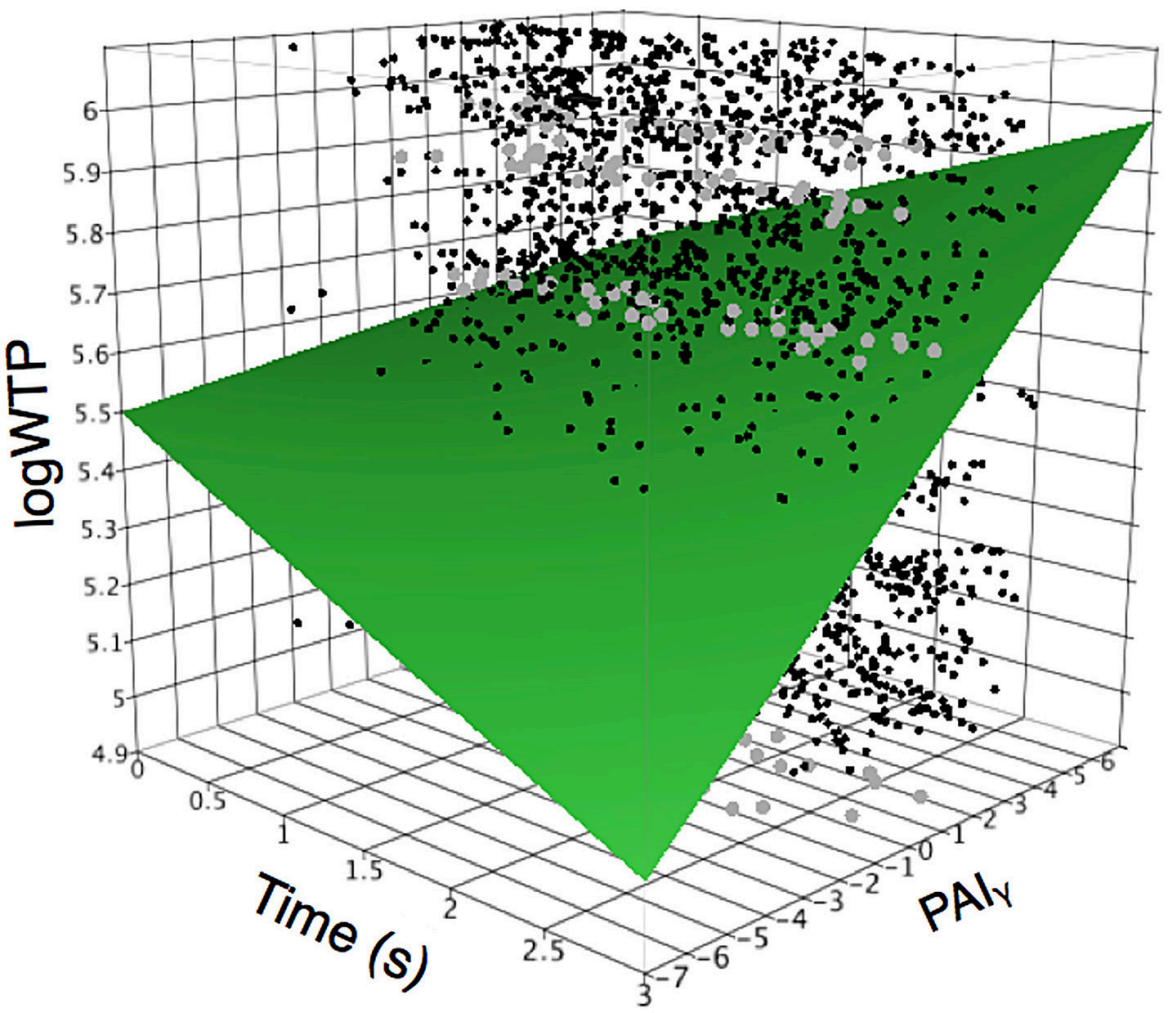
Interactions between time and gamma on relationship to WTP. Relationship between prefrontal gamma laterality (PAI_γ_) and time demonstrates that the laterality effect is strongest at the end of product viewing time, and smallest during the first period of product viewing. The plot shows the interrelationship between time from stimulus onset, PAI_γ_, and logWTP, and how the relationship between PAI_γ_ and logWTP changes with time. In particular, note that at the time of stimulus onset (time is low) the PAI_γ_ is only weakly positively related to logWTP, while at higher time score (stimulus has been displayed for longer time, and the participant is closer to making a choice) the relationship between PAI_γ_ and logWTP is dramatically more positive. Dots denote actual data points, where black dots represent single data points, and gray dots represent multiple converging data points.

Our final analysis tested whether the relationship between prefrontal asymmetry and WTP would be modulated over time. Our mixed model analysis testing the interaction between time and PAI scores for each frequency demonstrated a significant explanatory effect (*R*^2^ = 0.299, *p* < 0.0001) with significant interaction effects only for the gamma frequency (PAI_γ_, see Table 3). As shown in Figure 4, the relationship between PAI_γ_ and WTP was higher for longer stimulus durations, i.e., the closer subjects were to making the actual decision. To test whether the relationship between PAI_γ_ and WTP was significant already at stimulus onset, we ran a *post-hoc* mixed model analysis with WTP as the dependent variable, and with PAIγ during the first second as the independent variable and with subject as random factor. This showed that even during the first second of product viewing, PAI_γ_ was significantly related to WTP (*R*^2^ = 0.309, *F* = 41.2, *p* < 0.001). This explanatory value was better than 2 s into product viewing (*R*^2^ = 0.292, *F* = 9 5.2, *p* < 0.001), but less than the third second of product viewing (*R*^2^ = 0.315, *F* = 390.5, *p* < 0.001).

**Table 3:** Effects of interaction between frequency band and time.

**Source**	**DF**	**DFDen**	***F***	***p***
PAIα	1	53160	34.2827	<0.0001^*^
PAIβ	1	53163	104.5560	<0.0001^*^
PAIγ	1	53166	222.1251	<0.0001^*^
PAIα^*^Time	1	53151	1.8140	0.1780
PAIβ^*^Time	1	53151	0.4596	0.4978
PAIγ^*^Time	1	53152	26.1636	<0.0001^*^
Time	1	53152	20.2815	<0.0001^*^

*Interaction effects (denoted by asterisk) between PAI measures and time, showing that prefrontal asymmetry in the gamma range, but neither alpha or beta, interacts specifically with time*.

## Discussion

In this study, we have found that brain responses during product viewing are significantly related to the variation in consumers' subsequent willingness to pay for the same products. At the time that subjects viewed products, measurements of prefrontal asymmetry in brain activation accounted for 27.5% of the variation in subsequent WTP. Taking product category, which explained ~45% of the variation in WTP, into account, improved the model's explanatory value to 64.1% of the variation in WTP.

These results provide novel insights into the basic psychological processes underlying WTP calculations and consumer choice. First, we find that WTP is mainly explained by prefrontal asymmetry in the gamma frequency band, and tentatively in the beta band. Second, prefrontal asymmetry in the gamma oscillation band showed an improved explanatory relationship with WTP responses the closer a subject is to making the actual decision, yet the model is still significant during the first second of product viewing. Both these effects have significant implications for our understanding of the psychological mechanisms underlying WTP, and will be discussed accordingly in the following paragraphs. Finally, we discuss the extent to which these EEG measures of brain activation can be used as predictive value of consumer choice as scalable, commercial applications.

Before any further discussion of the results, three issues should be noted. First, this study had a sample size of only 16 participants, a relatively small sample, in which the power of the statistics is relatively low. Further replication is needed to ensure the replicability and external validity of this study.

Second, the current study only tested women, as part of a larger study on compulsive buying behaviors in women. Although this study only tested healthy, non-compulsive consumers, the results do not yet warrant a gender-free interpretation. Thus, more studies on frontal asymmetry and consumer choice should include both women and men, and explore potential differences in asymmetric responses.

Third, in this study, we employed the Emotiv EPOC low-cost EEG system. One question could be raised about potentially lower data quality of this headset. To this end, we show that the signal quality is acceptable for the purpose of this study. This corresponds with prior studies demonstrating that this headset produces acceptable data quality and that it reproduces brain responses (both event-related potentials and frequency based responses) comparable to what has been found in studies with higher-resolution systems (Badcock et al., 2013; Grummett et al., 2014; Christopher et al., 2015; Wang et al., 2015), including emotional processing (Harmon-Jones et al., 2010; Khushaba et al., 2013), and even in mobile settings (Allison et al., 2010; Debener et al., 2012). This said, further support from these findings is needed from studies using high-resolution EEG and other neuroimaging approaches.

### Effects of separate activity types

The present results provide novel insights into the mechanisms of prefrontal asymmetry and their relevance to consumer choice. Prefrontal EEG asymmetry, typically reported in the alpha-band range (PAI_α_), has been related to cognitive and emotional processes (Harmon-Jones and Allen, 1998; Pizzagalli et al., 2005; Ohme et al., 2009, 2010; Ravaja et al., 2012). Our results contradict these findings by showing that there was no significant relationship between frontal asymmetry in the alpha band and WTP decisions, even when this frequency was studied in isolation. This finding is significant, as it may suggest a specific nuance in the way that alpha band asymmetry should be interpreted in light of particular types of decision-making, and our understanding of the neurobiological mechanisms underlying the mental calculations necessary for reaching a final decision on what to pay for a specific product.

Alpha oscillations are classically related to activation decrease or inhibition of neural activation, even as an “idling rhythm” of the brain (Coan and Allen, 2003; Roche, 2004; Sauseng et al., 2005; Händel et al., 2011; Smith et al., 2017; van Diepen and Mazaheri, 2017). However, recent reports have also implied a more complex functional role of the alpha band, such as divergent thinking (Benedek et al., 2011), tonic alertness (Sadaghiani et al., 2010), and mirror neuron function (Oberman et al., 2005). Notably, Sabate et al. (2012) reported a dual nature of alpha oscillations with respect to attention, in that alpha was both related to attentional boosting of selected task calculations while decreasing the computation of other potentially interfering tasks. In the present context of prefrontal asymmetry, prior studies have linked changes in prefrontal alpha band asymmetry to approach behaviors, including reward expectancy and decision making (Miller and Tomarken, 2001), and individual traits such as reward sensitivity (Pizzagalli et al., 2005) and obesity (Ochner et al., 2009). Taken together, this suggests that the change in PAI_α_ may be related to multiple roles throughout the period of product viewing, including approach behavior and attentional gating, but not necessarily something that is crucial for making value-based decisions that manifest as WTP decisions. Notably, as PAI_α_ was not affected by product viewing time, it is possible that frontal asymmetry in the alpha frequency band is not involved in the calculations of here-and-now calculations of product value, neither at the immediate evaluation level, or during choice execution. Our findings thus challenges and nuances research on frontal alpha asymmetry and approach behavior by demonstrating that it is not related to particular choice behaviors, and by suggesting that further studies are needed to delineate the link between the neurobiology of approach behavior and consumer choice.

A novel finding was that prefrontal asymmetry in the gamma range, and tentatively in the beta range, was significantly related to WTP choices. Prefrontal asymmetry in the beta range (PAI_β_) was only trend significant and thus brings a low explanatory value in relating to WTP, yet provides interesting and significant trends that should be explored further. To our knowledge, this is also the first demonstration of in PAI_β_ in value-based decision-making. Previous studies have demonstrated a link between beta frequency and subjective preference, such as Boksem and Smidts (2015), in which frontal beta during movie trailers were found to be significantly correlated with subsequent individual preference judgments. However, here, as in other studies of beta and choice (Polanía et al., 2014; Chand et al., 2016; Jo et al., 2016), no frontal beta asymmetry was reported, and was not part of the aims of the study. In this study, PAI_β_ demonstrated a negative relationship with WTP. This suggests that stronger left vs. right prefrontal asymmetry is related to a lower subsequent willingness to pay for a product. Despite the relative small size of this effect, this finding is unexpected and warrants further studies. Beta band activation in general has been linked to somatosensory and somatomotor functions (Cebolla et al., 2009; Ritter et al., 2009), language processing (Spironelli and Angrilli, 2010; Wang et al., 2012; Weiss and Mueller, 2012), and in complex decision-making and every-day behavior (Davis et al., 2011). Notably, beta oscillations have recently been implicated in perceptual decision making. For example, by using local field potential EEG in monkeys while they performed a comparison decision task, Haegens et al. (2011) found that beta oscillations during a evaluation and comparison phase were related to subsequent choice behavior. This may suggest a role for beta oscillations in comparing choice options, and that prefrontal asymmetries in this frequency band are related to value-based consumer choices. However, the observed effects in our own data showed unexpected features such as an inverse prefrontal asymmetry effect and no effect of viewing time. Interestingly, PAI_β_ did not change with higher proximity to the choice execution. This is at odds with recent studies that have linked beta to the decision to move (Jo et al., 2016). Also, a recent study by Boksem and Smidts (2015) showed that general beta synchrony was predictive of consumer preference and subsequent choice, suggesting a general frontal beta engagement in consumer choice. However, such research has pointed to beta being more related to a meta-cognitive aspect of decision-making (Chand et al., 2016), and for frontal theta, see Wokke et al. (2017). As the aim of the present study was to study the asymmetric frontal engagement of the brain, it is not an appropriate model for studying general beta-related activity related to the time of choice. Consequently, more studies are needed to understand the function of prefrontal beta oscillations with respect to consumer choice.

A substantial prefrontal asymmetry effect was found in the gamma oscillation band (PAI_γ_). Gamma synchrony is thought to represent a specific kind of activation, and is believed to play an important role in the synchronization within functional units, integrating proximate or distant functional units, and can do so in a time-locked or phase-locked manner (Başar et al., 1999). Gamma activation has been shown to relate to a number of cognitive processes, including object recognition (Schadow et al., 2009; Castelhano et al., 2014; Ahlfors et al., 2015), memory types (Başar et al., 1999, 2001; Nyhus and Curran, 2010; Roux and Uhlhaas, 2014; Heusser et al., 2016; Després et al., 2017), and conscious processing (Aru and Bachmann, 2009; Doesburg et al., 2009; Luo et al., 2009; Steinmann et al., 2014; Cabral-Calderin et al., 2015; Tu et al., 2016). Most notably, gamma has been linked to the functional coupling—or binding—of brain regions that ensures integration and appropriate processing of information (Klimesch et al., 2010; Ehm et al., 2011; Schneider et al., 2011). This may suggest that the observed effects of PAI_γ_ on WTP may be related to a specific kind of value computation and possibly the link to choice execution. Notably, in a study by Ravaja et al. (2012), frontal asymmetry was found to predict consumer choice in the face of changes in price and brand provided. However, as this asymmetry was focused on alpha, we believe that our study is the first demonstration of a role for frontal asymmetric gamma oscillations in consumer choice. Moreover, as this study was for a particular type of value computation—the specific monetary valuation of products. Thus, these findings show that, besides not confirming the traditional asymmetry index in the alpha range, gamma is strongly related to consumption behavior, thus suggesting specific psychological mechanisms (for gamma and to some extent beta) in WTP calculations.

An exploratory whole-brain analysis was conducted to test for other types of EEG responses related to WTP, and the results are shown in Table 4.

**Table 4:** Whole-brain analysis of alpha, beta, and gamma synchrony and WTP.

**Channel/frequency**	**Estimate**	**Std. Error**	**df**	**T**	***p***
**ALPHA**					
AF3	0.00004	0.00013	9,865	0.31	0.7593
AF4	−0.00035	0.00012	9,866	−2.80	**0.0051**
F3	−0.00059	0.00017	9,863	−3.54	**0.0004**
F4	0.00156	0.00029	9,867	5.46	**<0.0001**
F7	0.00025	0.00019	9,864	1.31	0.1918
F8	0.00008	0.00006	9,862	1.39	0.1635
FC5	0.00040	0.00043	9,867	0.92	0.358
FC6	−0.00020	0.00040	9,868	−0.49	0.6227
O1	0.00012	0.00066	9,867	0.18	0.8557
O2	0.00043	0.00021	9,870	2.08	**0.0373**
P7	−0.00117	0.00155	9,869	−0.76	0.4482
P8	−0.00016	0.00003	9,870	−4.58	**<0.0001**
T7	−0.00001	0.00041	9,874	−0.03	0.9731
T8	0.00001	0.00003	9,872	0.22	0.828
**BETA**					
AF3	−0.00003	0.00015	9,844	−0.18	0.8533
AF4	−0.00021	0.00014	9,847	−1.48	0.1383
F3	−0.00023	0.00011	9,842	−2.00	**0.0462**
F4	0.00032	0.00049	9,846	0.65	0.5159
F7	0.00027	0.00021	9,843	1.27	0.2031
F8	0.00000	0.00004	9,842	−0.03	0.9784
FC5	0.00008	0.00031	9,843	0.27	0.7897
FC6	−0.00036	0.00054	9,844	−0.66	0.5074
O1	0.00052	0.00052	9,845	0.99	0.3208
O2	−0.00107	0.00042	9,851	−2.56	0.0105
P7	−0.00458	0.00133	9,846	−3.45	**0.0006**
P8	0.00021	0.00004	9,847	5.21	<0.0001
T7	−0.00003	0.00088	9,844	−0.03	0.9745
T8	0.00019	0.00006	9,848	3.24	**0.0012**
**GAMMA**					
AF3	0.00125	0.00027	9,873	4.73	**<0.0001**
AF4	−0.00014	0.00024	9,866	−0.60	0.5472
F3	0.01580	0.00140	9,868	11.25	**<0.0001**
F4	−0.00191	0.00117	9,876	−1.63	0.1024
F7	0.00128	0.00046	9,865	2.81	**0.005**
F8	−0.00354	0.00129	9,868	−2.74	**0.0062**
FC5	−0.00145	0.00115	9,868	−1.25	0.2101
FC6	−0.00242	0.00082	9,868	−2.96	**0.0031**
O1	−0.04786	0.00613	9,873	−7.80	**<0.0001**
O2	−0.00329	0.00314	9,877	−1.05	0.2943
P7	0.00052	0.00282	9,867	0.19	0.8524
P8	−0.00001	0.00001	9,866	−2.47	**0.0135**
T7	−0.00005	0.00009	9,870	−0.54	0.5865
T8	−0.00049	0.00025	9,879	−1.95	0.0515

*Exploratory whole-brain analysis of the relationship between frequency band power for each of the electrodes and logWTP, using a mixed model analysis. Statistical significant values with a p < 0.05, uncorrected, are highlighted with bold text*.

### Frontal asymmetry and product category

Not surprisingly, since we tested products such as FMCG products and luxury goods, WTP was significantly affected by product category. However, a notable observation was that the explanatory power of frontal asymmetry in different frequency bands were affected when product category was used as a regressor in the analysis. First, asymmetry in the beta frequency was unaffected by product category, and the main effect of this frequency became insignificant (not even a trend) when product category was included as a covariate in the regression model. This suggests that asymmetry in the beta range is possibly even less important in WTP evaluations and choice, and caution should be emphasized when interpreting even the main effect for beta frequency asymmetry.

Conversely, frontal asymmetry in the gamma range was significantly influenced by product category. Bags and shoes showed a significant positive relationship to WTP, in that higher asymmetry scores (stronger left than right asymmetric engagement) were associated with a higher willingness to pay for the product. For clothing, no significant relationship was found. Interestingly, we find that the relationship between frontal gamma asymmetry and WTP was significantly negative for FMCG products. That is, the higher the asymmetry (denoting a stronger left than right engagement) the lower the WTP.

Taken together, this suggests that emotional responses during product viewing show a product-specific relationship to what we are willing to pay for a product. With fashion items and products associated with “conspicuous consumption” (Kastanakis and Balabanis, 2014; Wang and Griskevicius, 2014), it may be less of a surprise that more positive emotional responses are related to a higher price point for the product (and thus possibly a lower price sensitivity). Conversely, for everyday FMCG products, higher asymmetry scores were related to a lower willingness to pay, suggesting that these products have a very different price sensitivity. However, as this study did not actively look for these types of responses, further research need to study these effects more specifically, as well as address the nature of the WTP-PAI relationship, and to the extent it is linear or show more complex non-linear properties. Research should also be conducted on groups who have different levels of interest for the products tested. In this study, we cannot rule out that the strong positive relationship between frontal asymmetry and WTP for bags and shoes is driven by our selection of women who were recruited for testing these types of products. Thus, additional studies on different consumer segments and varying product interests are needed.

### Frontal asymmetry and proximity to decision-making

The assertion of a specific and independent role of prefrontal gamma asymmetry is further corroborated by our final analysis, in which we tested the interactions between PAI scores in all three frequency bands and product viewing time. Here, our data showed that the relationship between PAI_γ_ and WTP was significantly modulated by time. In particular, the closer to the actual decision, the stronger the relationship. No such effect was found for the PAI_α_ or PAI_β_, which possibly implies a role for frontal asymmetry in the beta frequency range in more stable value calculations, and a dissociation from choice execution. This finding is closely related to recent studies demonstrating a role of the dlPFC in planning and executing the actual choice, while other regions such as the OFC and ACC are more related to the initial valuation of the choice options (Petrides and Pandya, 1999; Rolls, 2000, 2004; Plassmann et al., 2007, 2010; Rushworth et al., 2012). Future studies should seek to combine imaging techniques, such as simultaneous fMRI and EEG (Moosmann et al., 2008; Rosa et al., 2010), to reveal the specific morphological and neuropsychological nature of the different oscillations and their roles over time.

### Prefrontal asymmetry and consumer engagement, motivation, and choice

The present finding is among the first to demonstrate a significant relationship between brain activity and willingness to pay in consumer choice. Indeed, in contrast to a study on the brain basis of WTP using fMRI (Plassmann et al., 2007), our approach demonstrates the added value of EEG as an imaging modality for assessing consumer preference and choice. In addition to the insights gained from this on the basic mechanisms of consumer choice, our results also answers calls for neuroimaging measures that assess and predict of consumer response and choice (Butler, 2008; Garcia and Saad, 2008; Murphy et al., 2008; Senior and Lee, 2008; Wilson et al., 2008; Ariely and Berns, 2010; Fisher et al., 2010; Lee et al., 2017). Indeed, great strides are currently made in the application of neuroimaging tools and neuroscience insights in understanding, measuring and affecting consumer choice. As the demand for measures of subconscious emotional and cognitive responses is currently peaking, and substantially higher than other research approaches (http://www.greenbookblog.org/2017/06/27/the-top-20-most-in-demand-supplier-types-at-iiex-na-2017/) it is crucial that such measures being used are thoroughly documented, validated and applied to the relevant contexts. Here, it is likely that the indexing of prefrontal asymmetry responses may hold predictive powers of consumer choice in similar as well as other contexts. Even so, studies have recently demonstrated that even in a relatively small sample, brain responses can predict not only individual choice, but even market effects, such as music hits, twitter feeds, TV ratings, and box office movie sales (Berns and Moore, 2012; Dmochowski et al., 2014; Boksem and Smidts, 2015), further supporting the idea that neuroscience can provide substantial added value to consumer research, both academically and commercially. This warrants further studies, and a few notable questions should be addressed in future research:

- What are the relationship between PAI and WTP when the duration between assessment and choice is prolonged, as in when there are hours, days, and even weeks and months between the PAI assessment and consumer choice?
- What is the relationship between frontal asymmetry in different frequency bands (alpha, beta, gamma), and how do they relate to different types of value based decision-making?
- Which brain structures are most involved in the separate effects found for prefrontal alpha, beta, and gamma oscillations? Do they represent separate mechanisms of choice in, brain regions such as the OFC, dlPFC, and ACC?
- Does the effect of time on PAI_γ_ indicate separate neural mechanisms, such as OFC during the early face and dlPFC during the late phase? Is the PAI_γ_ a carrier of information from the product evaluation point to the choice execution?
- What is the temporal unfolding of frontal asymmetry, as measured by other types of EEG analyses, such as Event-Related Potentials (ERPs)?
- What is the predictive value of frontal asymmetry on larger market effects? Is frontal asymmetry more related to individual choice, or does it also signify coherent human responses at a cultural level?

## Ethics statement

This study was carried out in accordance with the recommendations of the Copenhagen Ethics Committee with written informed consent from all subjects. All subjects gave written informed consent in accordance with the Declaration of Helsinki. The study was approved under the ethical protocol KF 01–131/03, issued by the local ethics committee.

## Author contributions

TR was the PI and worked on study design, project management, data preprocessing, data analysis, and was main responsible for the manuscript; MS worked on the study design and results interpretation, and on manuscript writing; MC worked on data collection and data preprocessing, and contributed to the manuscript; CS worked on study design, data preprocessing and analysis, and contributed to the manuscript.

### Conflict of interest statement

The authors declare that the research was conducted in the absence of any commercial or financial relationships that could be construed as a potential conflict of interest.

## Abbreviations

EEG: electroencephalography
WTP: Willingness to Pay
PAI: Prefrontal Asymmetry Index.
